# Epidemic Catarrhal Influenza

**Published:** 1882-01

**Authors:** 


Epidemic Catarrhal Influenza.—Exaggerated and sensational reports have been appearing in the daily papers concerning the epidemic which has been prevailing the last two months. The so-called mysterious "pink-eye" malady is nothing more than a catarrhal epidemic influenza, very common among horses at all times, but more frequent and severe during damp and changeable weather. The sudden atmospheric changes of the present fall have been of a character particularly favorable to the development of this disease, which has in most instances assumed a mild type. The sequelæ, however, have been of a more grave character and of a different type than we have had in some years.

The tendency on the part of some of our veterinarians to encourage the scare and misrepresent the actual character of the disease is certainly reprehensible. Let us have greater effort in the direction of a more accurate knowledge of diseases and greater skill in treating them, rather than ambition to get before the public, no matter how or by what process.

